# Supplementary material for: Common gene expression signatures in Parkinson’s disease are driven by changes in cell composition
Source: Acta Neuropathol Commun. 2020 Apr 21;8:55. doi: 10.1186/s40478-020-00932-7 (PMC7175586; doi:10.1186/s40478-020-00932-7)
Supplement: Supplementary file 2 — Additional file 2: Figure S1. Read mapping efficiency; Figure S2. Read mapping statistics; Figure S3. Sample clustering; Figure S4. Neuronal MGPs and expression of neuronal markers; Figure S5. Oligodendrocyte MGPs and expression of oligodendrocyte markers; Figure S6. Microglial MGPs and expression of microglial markers; Figure S7. Astrocyte MGPs and expression of astrocyte markers; Figure S8. Endothelial MGPs and expression of endothelial markers; Figure S9. Neuroexpresso neuronal markers and overlap with single-cell neuronal DEGs; Figure S10. Cellular estimates grouped by status; Table S1. Correlations between MGPs calculated on different marker sets. [file 40478_2020_932_MOESM2_ESM.docx]

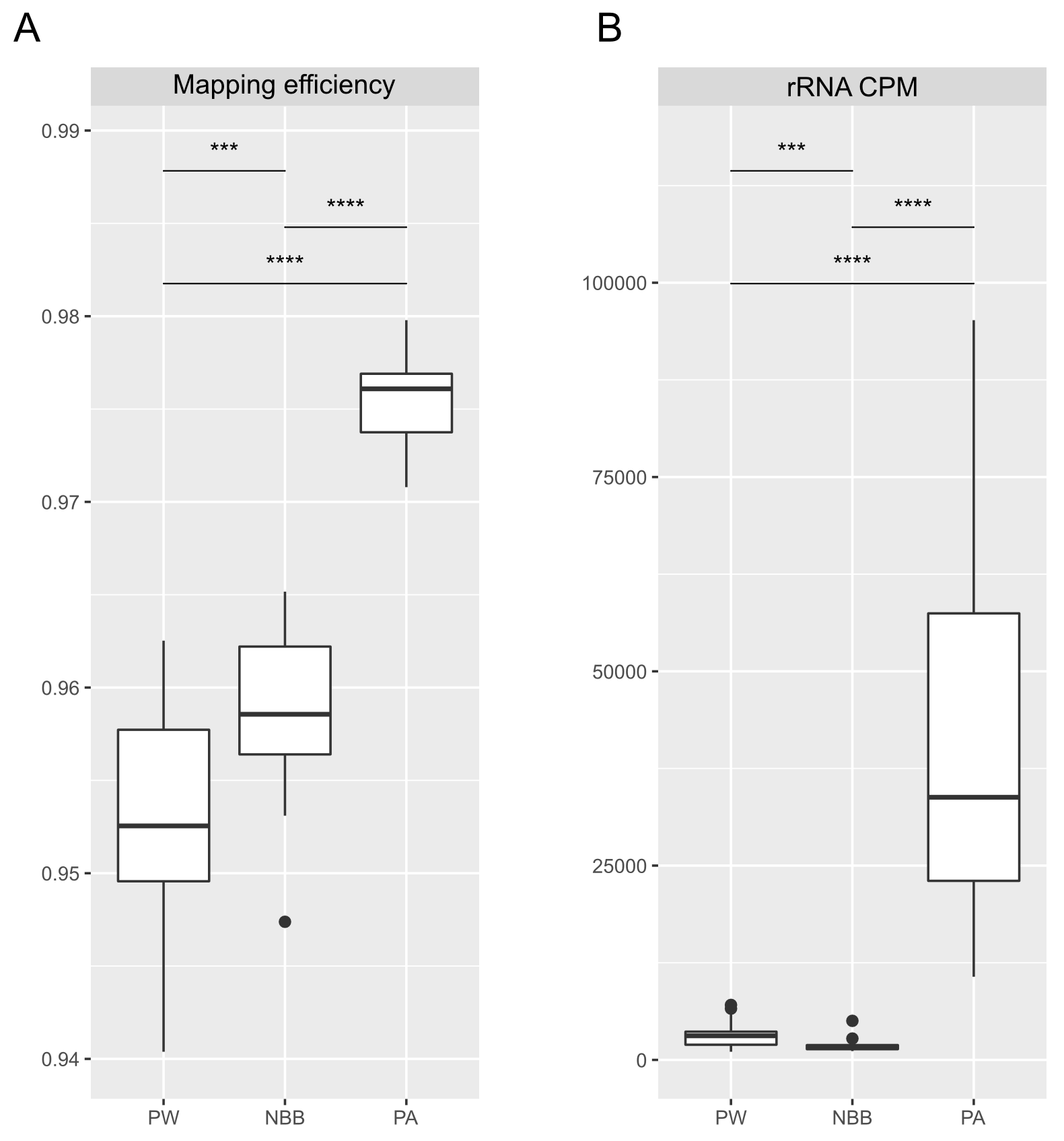


**Figure S1 – Read mapping efficiency**

(A) Proportion of reads uniquely aligned to the genome. (B) Counts per million (CPMs) for rRNA sequences (PW: ParkWest, NBB: Netherlands Brain Bank, PA: poly(A) capture cohort). Asterisks indicate significance at (*) p > 0.05, (**) p ≤ 0.01, (***) p ≤ 0.001, (****) p ≤ 0.0001, Wilcoxon test


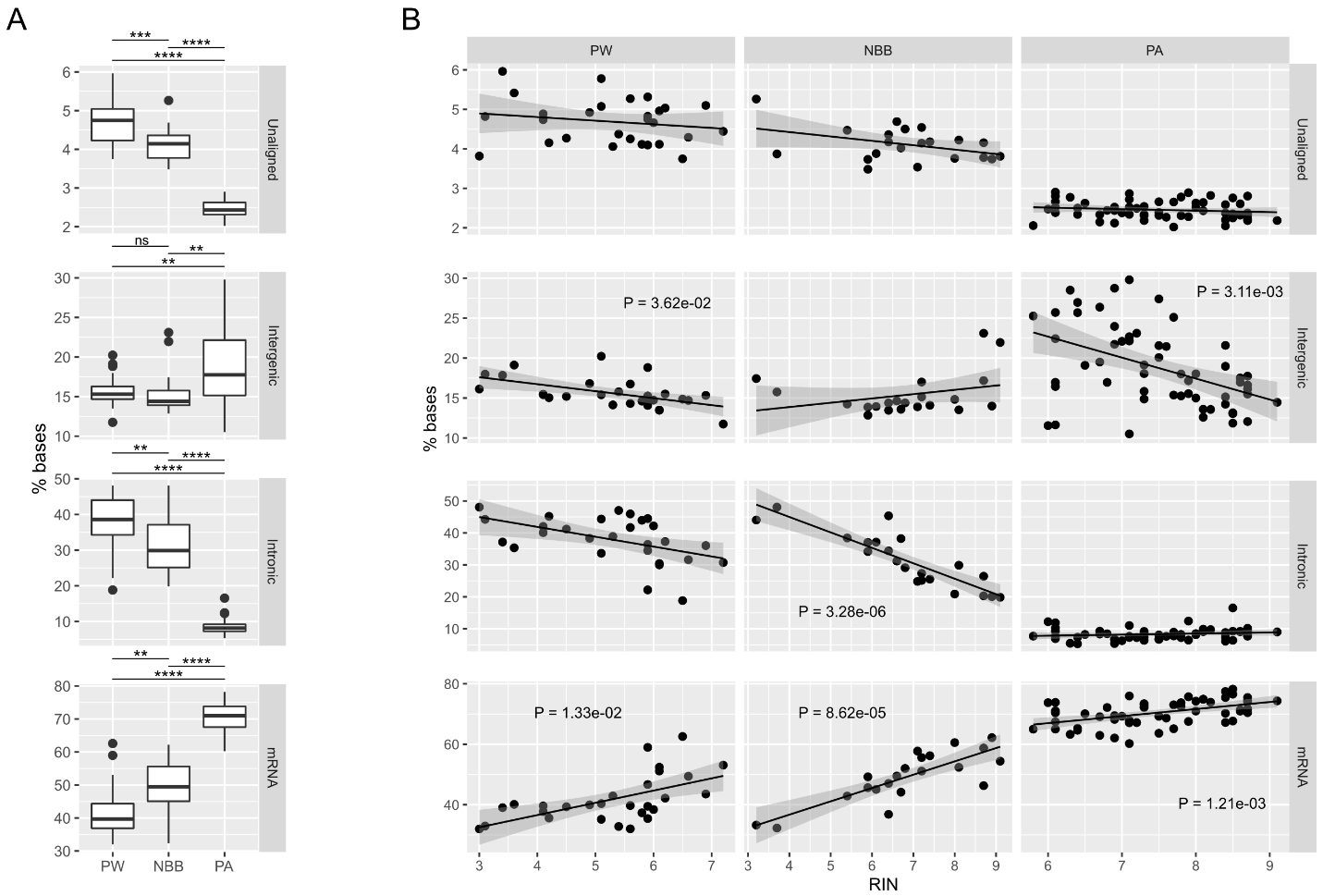


**Figure S2 – Read mapping statistics**

(A) Percentage of bases mapping to different genomic regions for the two cohorts analyzed using rRNA depletion (PW: ParkWest, NBB: Netherlands Brain Bank) and the poly(A) selection cohort (PA). (B) Scatterplots of samples show RIN values versus percent of bases (unaligned, intergenic, intronic and mRNA). Linear regression trend represented by a black line. Only significant linear regression p-values (p < 0.05) are shown in the panels (PW: ParkWest, NBB: Netherlands Brain Bank, PA: poly(A) capture kit). Asterisks indicate significance at (*) p > 0.05, (**) p ≤ 0.01, (***) p ≤ 0.001, (****) p ≤ 0.0001, Wilcoxon test


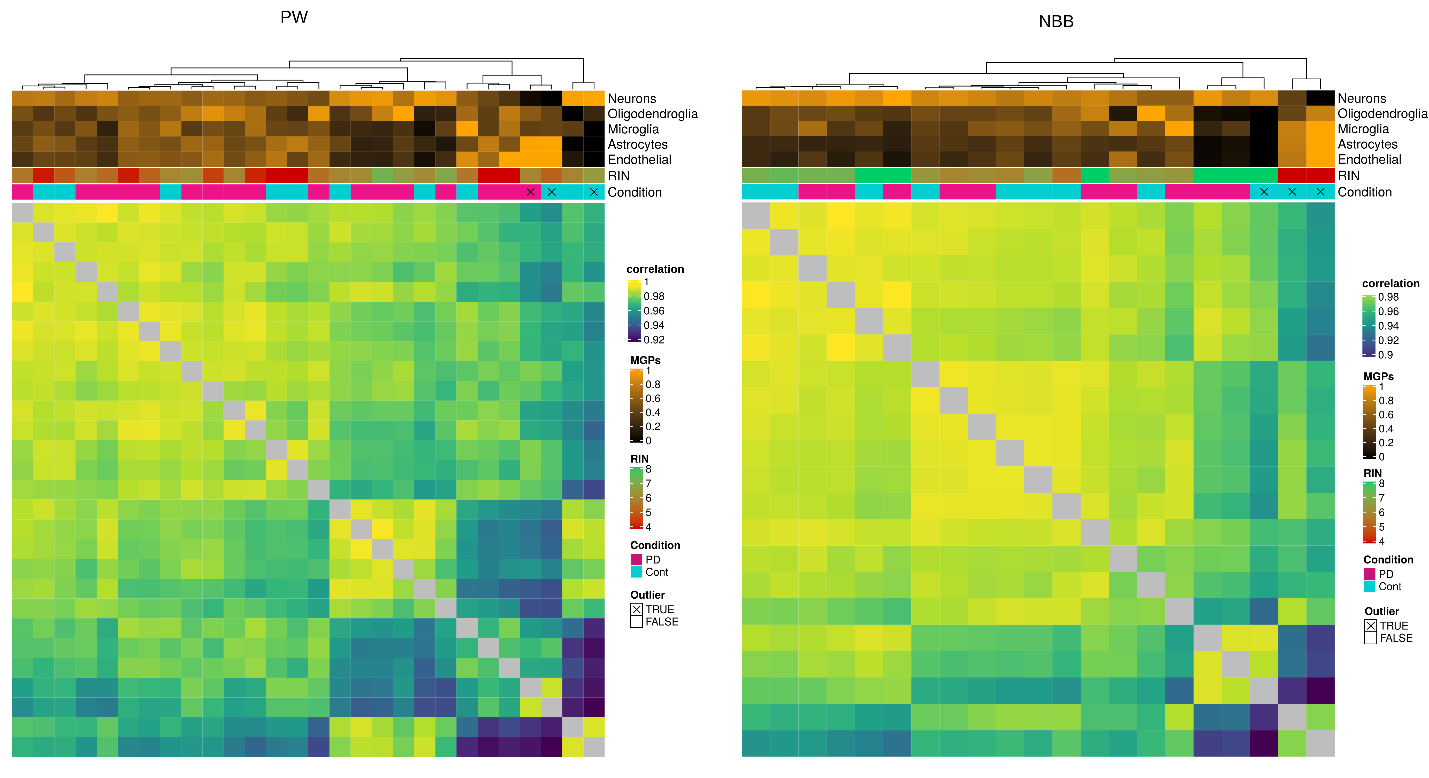


**Figure S3 – Sample clustering**

Samples were marked as outliers if the median correlation with the other samples was below Q1-1.5*IQR or above Q3+1.5*IQR, where IQR stands for inter-quartile range. For each of the Ribo-Zero cohorts, the heatmaps represent the pairwise correlation coefficients between the samples based on the log CPM. MGP estimates for the main cortical cell types are represented on the top of each heatmap, together with RIN, disease status, and the outlier status for each sample. PW: ParkWest, NBB: Netherlands Brain Bank**
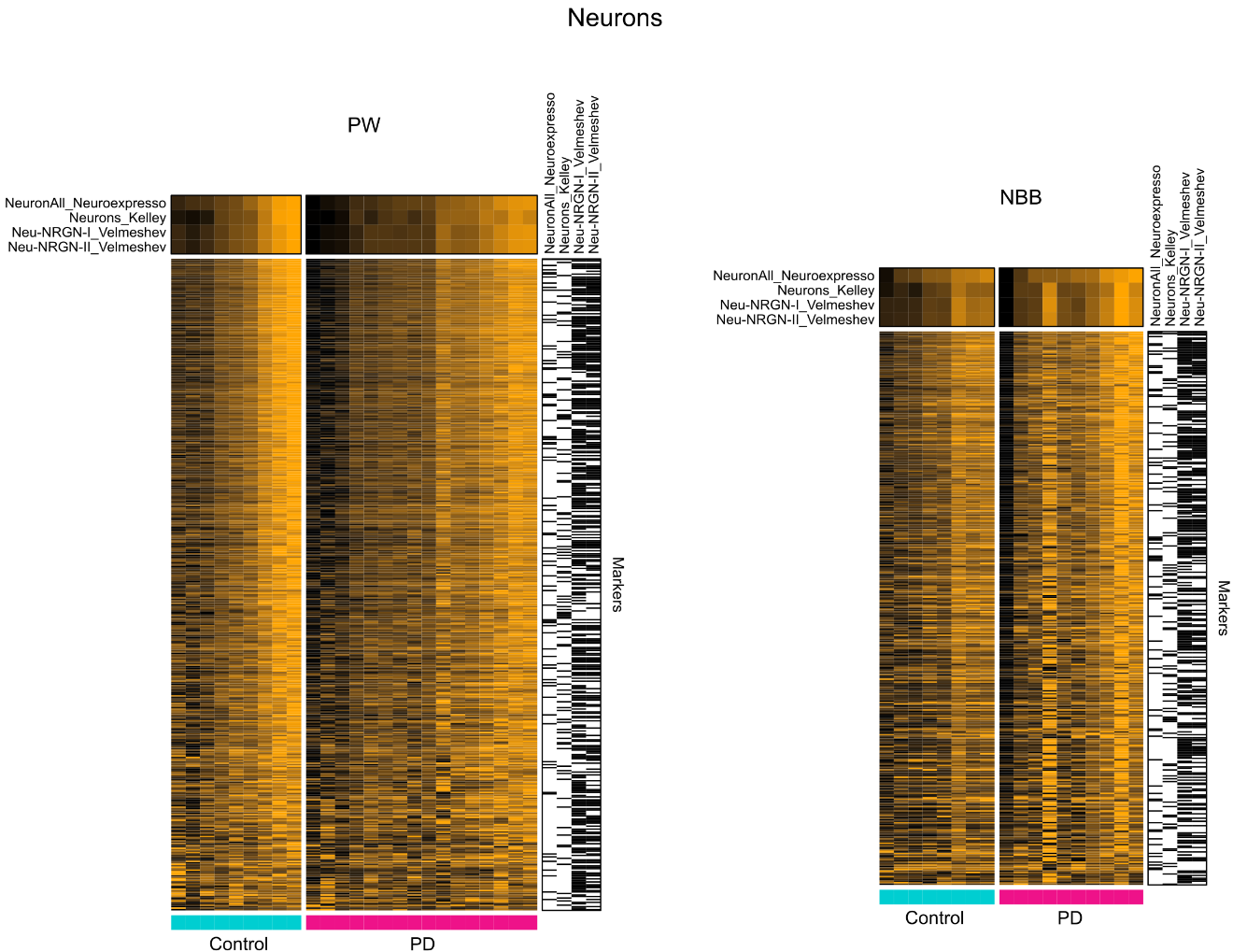
**

**Figure S4 – Neuronal MGPs and expression of neuronal markers**

Heatmaps represent the scaled expression (log(CPM + 1)) for each neuronal marker gene and each sample. Samples (columns) are grouped by condition (Controls and PDs) and sorted within each group by their MGP value. Marker genes (rows) are displayed if present in at least one of the marker sets in at least 80% of the permutations (NeuroEspresso markers, Mancarci et al. 2017 [1], Kelley et al. 2018 [2], Velmeshev et al. 2019 [3]). Since markers are not necessarily overlapping across the three sources, the memberships of the markers to a specific study are indicated in the right-hand side annotation (black = present in the marker set; white = absent from the marker set). The top rows in each heatmap represent the per-sample neuronal MGP values (one row for each marker set). PW: ParkWest, NBB: Netherlands Brain Bank

**
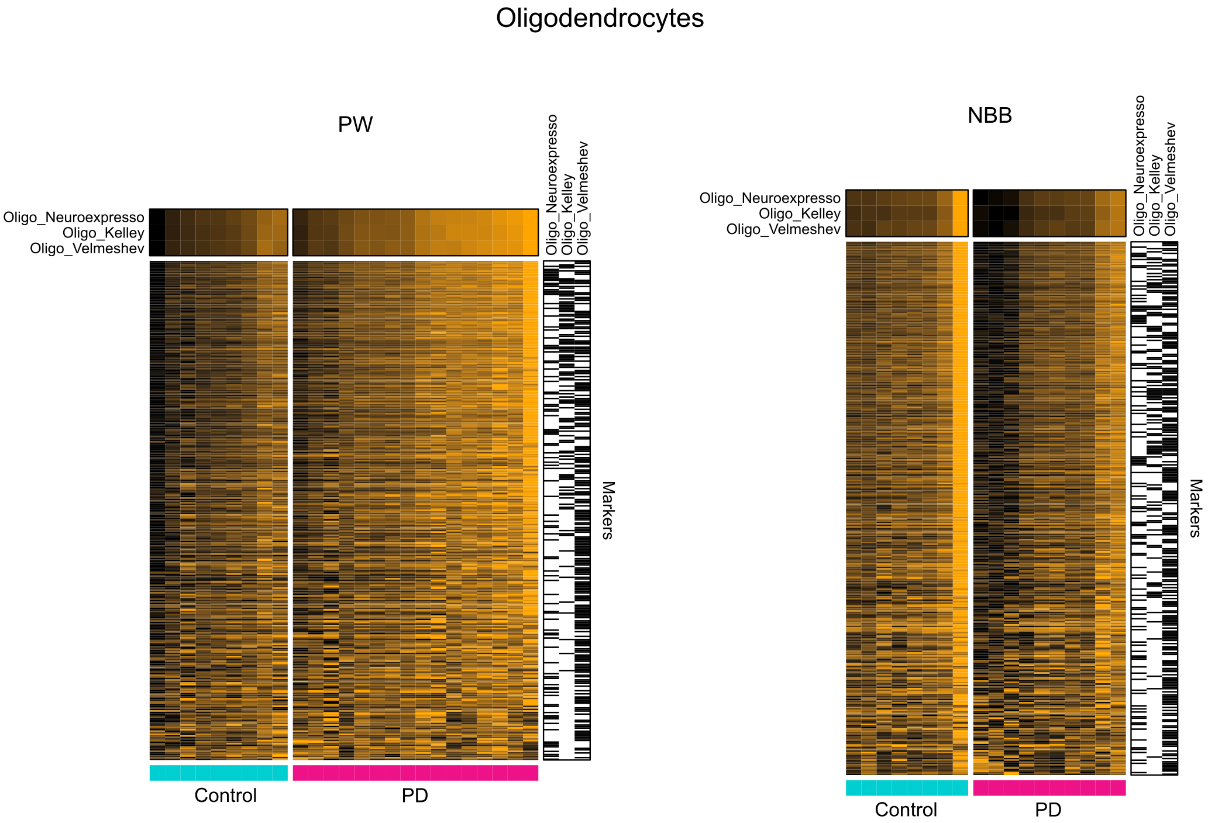
**

**Figure S5 – Oligodendrocyte MGPs and expression of oligodendrocyte markers**

Heatmaps represent the scaled expression (log(CPM + 1)) for each oligodendrocyte marker gene and each sample. Samples (columns) are grouped by condition (Controls and PDs) and sorted within each group by their MGP value. Marker genes (rows) are displayed if present in at least one of the marker sets in at least 80% of the permutations (NeuroEspresso markers, Mancarci et al. 2017 [1], Kelley et al. 2018 [2], Velmeshev et al. 2019 [3]). Since markers are not necessarily overlapping across the three sources, the memberships of the markers to a specific study are indicated in the right-hand side annotation (black = present in the marker set; white = absent from the marker set). The top rows in each heatmap represent the per-sample oligodendrocyte MGP values (one row for each marker set). PW: ParkWest, NBB: Netherlands Brain Bank

**
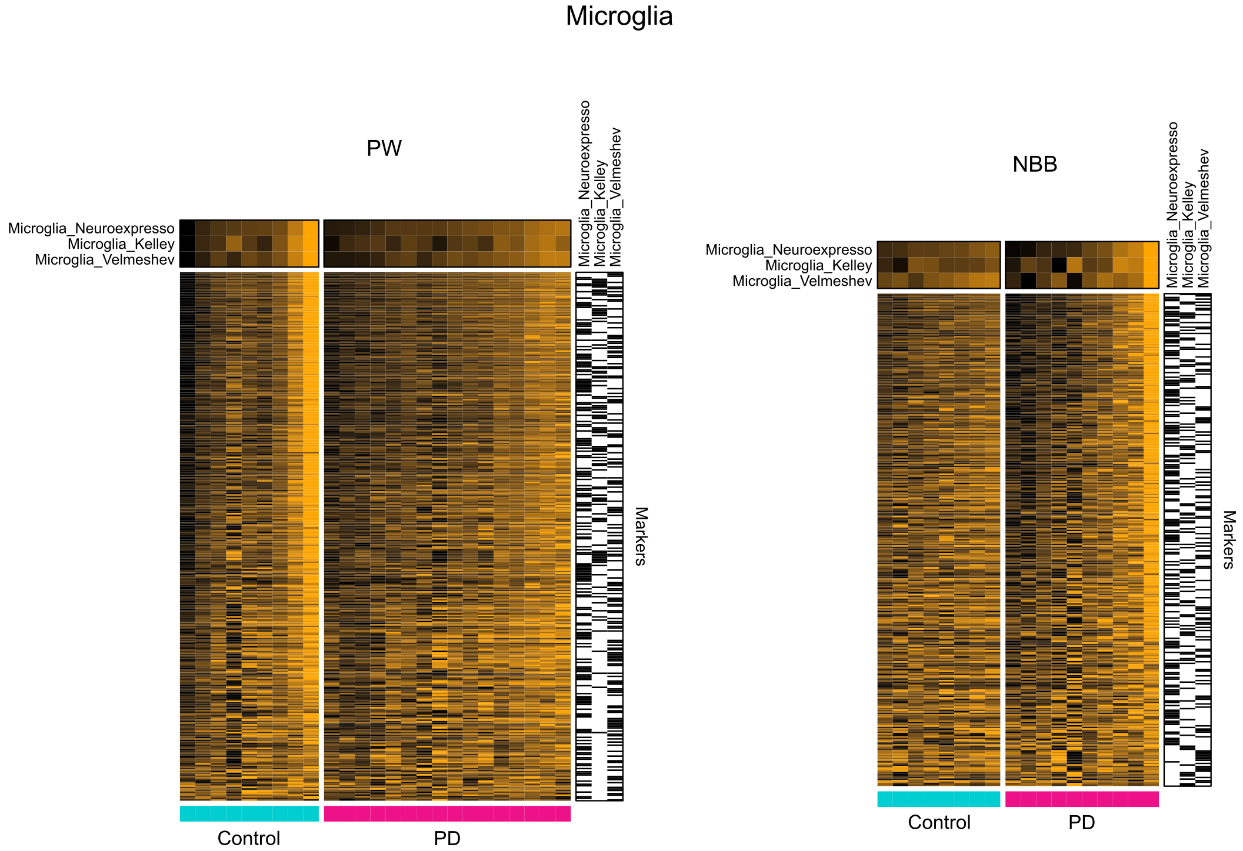
**

**Figure S6 – Microglial MGPs and expression of microglia markers**

Heatmaps represent the scaled expression (log(CPM + 1)) for each microglial marker gene and each sample. Samples (columns) are grouped by condition (Controls and PDs) and sorted within each group by their MGP value. Marker genes (rows) are displayed if present in at least one of the marker sets in at least 80% of the permutations (NeuroEspresso markers, Mancarci et al. 2017 [1], Kelley et al. 2018 [2], Velmeshev et al. 2019 [3]). Since markers are not necessarily overlapping across the three sources, the memberships of the markers to a specific study are indicated in the right-hand side annotation (black = present in the marker set; white = absent from the marker set). The top rows in each heatmap represent the per-sample microglial MGP values (one row for each marker set). PW: ParkWest, NBB: Netherlands Brain Bank

**
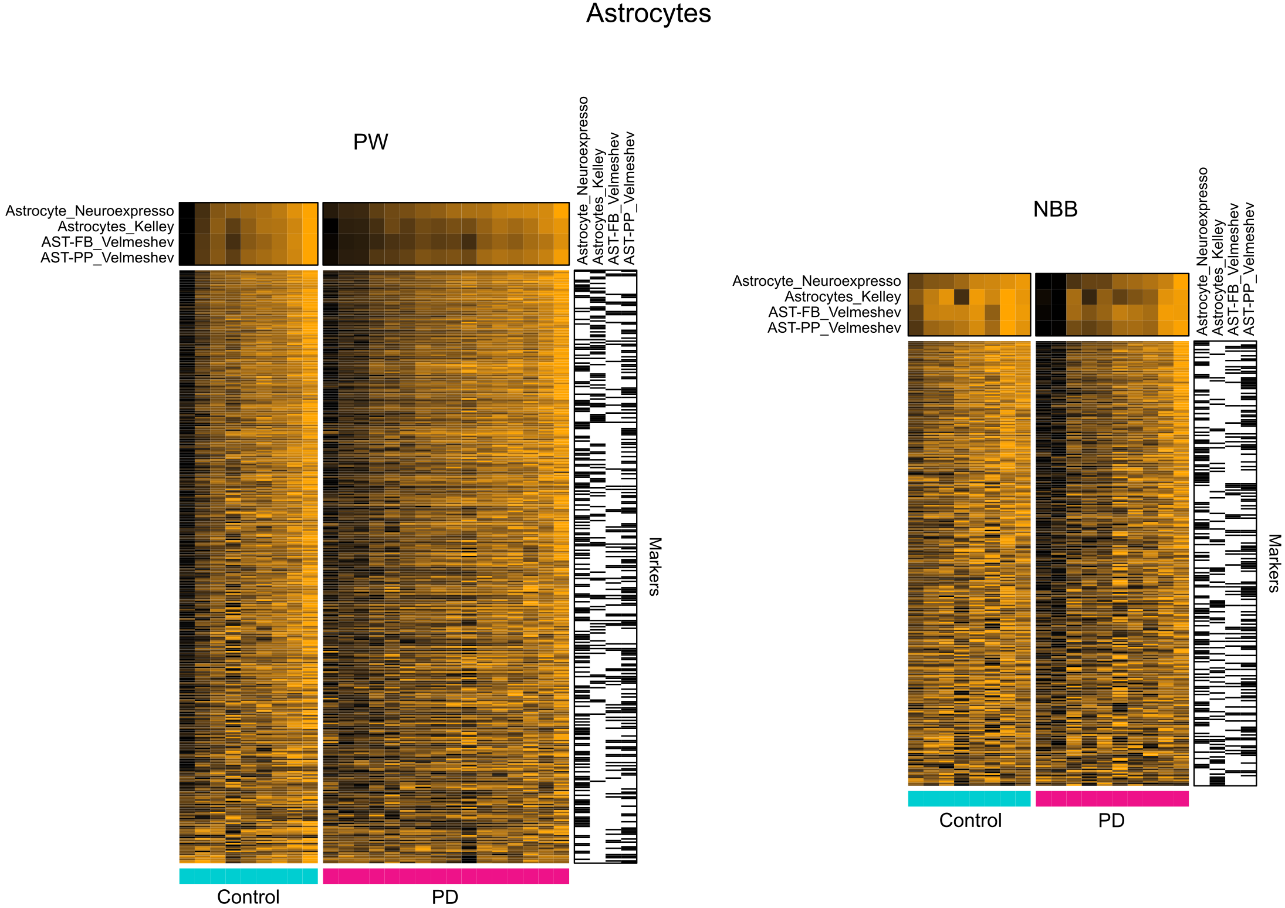
**

**Figure S7 – Astroctye MGPs and expression of astrocyte markers**

Heatmaps represent the scaled expression (log(CPM + 1)) for each astrocyte marker gene and each sample. Samples (columns) are grouped by condition (Controls and PDs) and sorted within each group by their MGP value. Marker genes (rows) are displayed if present in at least one of the marker sets in at least 80% of the permutations (NeuroEspresso markers, Mancarci et al. 2017 [1], Kelley et al. 2018 [2], Velmeshev et al. 2019 [3]). Since markers are not necessarily overlapping across the three sources, the memberships of the markers to a specific study are indicated in the right-hand side annotation (black = present in the marker set; white = absent from the marker set). The top rows in each heatmap represent the per-sample astrocyte MGP values (one row for each marker set). PW: ParkWest, NBB: Netherlands Brain Bank

**
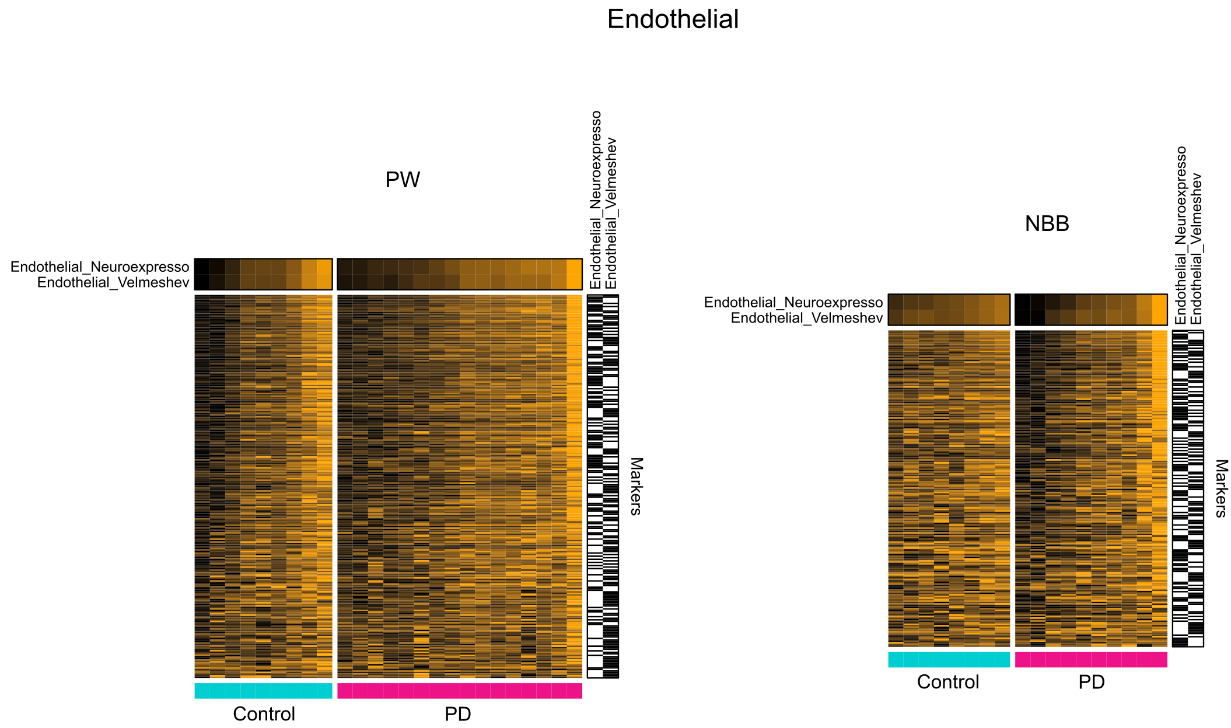
**

**Figure S8 – Endothelial MGPs and expression of endothelial markers**

Heatmaps represent the scaled expression (log(CPM + 1)) for each endothelial marker gene and each sample. Samples (columns) are grouped by condition (Controls and PDs) and sorted within each group by their MGP value. Marker genes (rows) are displayed if present in at least one of the marker sets in at least 80% of the permutations (NeuroEspresso markers, Mancarci et al. 2017 [1], Kelley et al. 2018 [2], Velmeshev et al. 2019 [3]). Since markers are not necessarily overlapping across the three sources, the memberships of the markers to a specific study are indicated in the right-hand side annotation (black = present in the marker set; white = absent from the marker set). The top rows in each heatmap represent the per-sample endothelial MGP values (one row for each marker set). PW: ParkWest, NBB: Netherlands Brain Bank


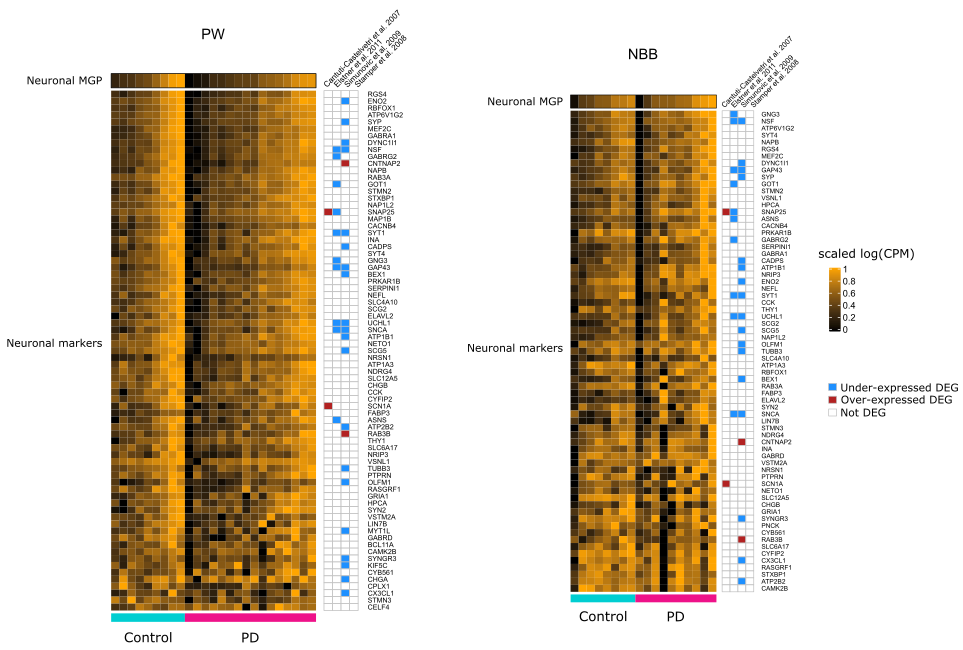


**Figure S9 – Neuroexpresso neuronal markers** **and overlap with single-cell neuronal DEGs in PD**

Heatmaps representing the scaled log(CPM) for Neuroexpresso neuronal markers (rows) selected in at least 80% of the permutations cross all samples in our cohorts (columns). Samples are grouped by condition within each cohort. Right-hand side annotations indicate differential expression of the markers in PD reported in LCM neurons [4–7]. Among the PD studies, only Stamper et al.2008 [5] corresponds to cortical neurons (blue = down-regulated, red = up-regulated). PW: ParkWest, NBB: Netherlands Brain Bank


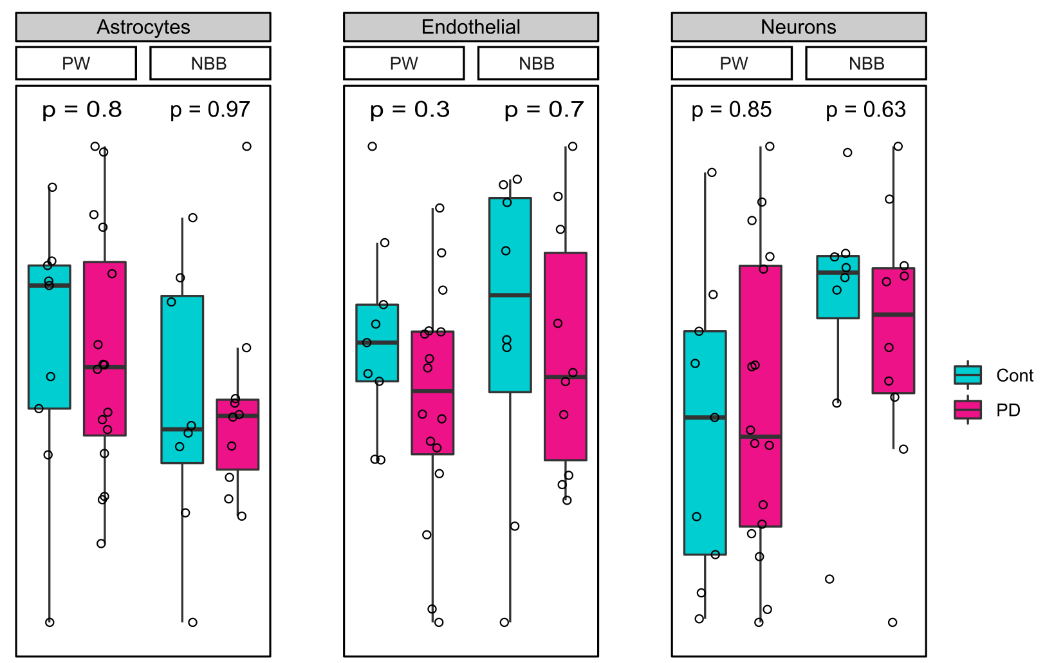


**Figure S10 - Cellular estimates grouped by status**

Astrocyte, endothelial, and neuronal MGPs adjusted for all the experimental variables (sex, age, PMI, RIN, and sequencing batch) and oligodendrocyte and microglia MGPs, but not for disease status. PW: ParkWest, NBB: Netherlands Brain Bank. P-values calculated with Wilcoxon test

**Table S1 - Correlations between MGPs calculated on different marker sets**

|  | **R** | **P** | **Overlap** |
| --- | --- | --- | --- |
| **Neuronal markers** |  |  |  |
| Neurons (Kelley *et al*. 2018) | 0.94 | 4.61x10^-21^ | 0.05 |
| NRGN-expressing neurons I (Velmeshev *et al*. 2019) | 0.96 | 1.14 x10^-23^ | 0.58 |
| NRGN-expressing neurons II (Velmeshev *et al*. 2019) | 0.97 | 4.46 x10^-26^ | 0.62 |
| **Oligodendrocyte markers** |  |  |  |
| Oligodendrocytes (Kelley *et al*. 2018) | 0.99 | 5.55 x10^-37^ | 0.23 |
| Oligodendrocytes (Velmeshev *et al*. 2019) | 0.99 | 1.65 x10^-37^ | 0.27 |
| **Microglial markers** |  |  |  |
| Microglia (Kelley *et al*. 2018) | 0.84 | 2.17 x10^-12^ | 0.28 |
| Microglia (Velmeshev *et al*. 2019) | 0.96 | 8.31 x10^-24^ | 0.12 |
| **Astrocyte markers** |  |  |  |
| Astrocytes (Kelley *et al*. 2018) | 0.95 | 2.12 x10^-22^ | 0.35 |
| Fibrous astrocytes (Velmeshev *et al*. 2019) | 0.85 | 4.03 x10^-13^ | 0.18 |
| Protoplasmic astrocytes (Velmeshev *et al*. 2019) | 0.96 | 7.20 x10^-24^ | 0.17 |

Correlation between MGPs calculated using the NeuroEspresso markers [1] and two marker sets based on single-cell transcriptomics of human cortex [2,3]). For dataset [2], the top 100 genes with the highest cell type fidelity for each cell type were used (R = Pearson’s correlation coefficient, P = p-value associated to the correlation; Marker overlap = proportion of genes shared, i.e. number of genes shared between the pair of marker sets divided by the smallest set size)

**Additional references**

1. Mancarci BO, Toker L, Tripathy SJ, Li B, Rocco B, Sibille E, et al. Cross-Laboratory Analysis of Brain Cell Type Transcriptomes with Applications to Interpretation of Bulk Tissue Data. eNeuro. 2017;ENEURO.0212-17.2017.

2. Kelley KW, Nakao-Inoue H, Molofsky AV, Oldham MC. Variation among intact tissue samples reveals the core transcriptional features of human CNS cell classes. Nat Neurosci. 2018;21:1171–84.

3. Velmeshev D, Schirmer L, Jung D, Haeussler M, Perez Y, Mayer S, et al. Single-cell genomics identifies cell type-specific molecular changes in autism. Science. 2019;364:685–9.

4. Cantuti-Castelvetri I, Keller-McGandy C, Bouzou B, Asteris G, Clark TW, Frosch MP, et al. Effects of gender on nigral gene expression and parkinson disease. Neurobiol Dis. 2007;26:606–14.

5. Stamper C, Siegel A, Liang WS, Pearson JV, Stephan DA, Shill H, et al. Neuronal gene expression correlates of Parkinson’s disease with dementia. Mov Disord Off J Mov Disord Soc. 2008;23:1588–95.

6. Elstner M, Morris CM, Heim K, Bender A, Mehta D, Jaros E, et al. Expression analysis of dopaminergic neurons in Parkinson’s disease and aging links transcriptional dysregulation of energy metabolism to cell death. Acta Neuropathol (Berl). 2011;122:75–86.

7. Simunovic F, Yi M, Wang Y, Stephens R, Sonntag KC. Evidence for gender-specific transcriptional profiles of nigral dopamine neurons in Parkinson disease. PloS One. 2010;5:e8856.
